# A High-Quality Chromosome-Level Genome Assembly and Comparative Analyses Provide Insights into the Adaptation of *Chrysomya megacephala* (Fabricius, 1794) (Diptera: Calliphoridae)

**DOI:** 10.3390/biology14080913

**Published:** 2025-07-22

**Authors:** Dan Zhang, Liangliang Li, Junchao Ma, Jianfeng Jin, Chunli Ding, Qiang Fang, Jianjun Jin, Zhulidezi Aishan, Xuebo Li

**Affiliations:** 1 Characteristic Laboratory of Forensic Science in the Universities of Shandong Province, Shandong University of Political Science and Law, Jinan 250014, China; zhangdanioz2016@gmail.com (D.Z.); lill@sdupsl.edu.cn (L.L.); majunchao@sdupsl.edu.cn (J.M.); dingcl@sdupsl.edu.cn (C.D.); 15866779290@163.com (Q.F.); 15847226031@139.com (J.J.); 2 School of Criminal Justice, Shandong University of Political Science and Law, Jinan 250014, China; 3 College of Life Sciences, Xinyang Normal University, Xinyang 464000, China; jianfeng.jin1995@gmail.com; 4 College of Life Science and Technology, Xinjiang University, Urumqi 830017, China

**Keywords:** forensic entomology, genome annotation, comparative genomics, gene family evolution

## Abstract

**Simple Summary:**

Numerous blowfly species (Calliphoridae) play vital roles in forensic death investigations, accurately determining postmortem intervals, death locations, and causes. *Chrysomya megacephala*, the most prevalent species in this field, exemplifies this capability. Nevertheless, critical gaps persist in chromosome-scale genomic data for *C. megacephala*, impeding comprehensive analysis of its biological mechanisms and evolutionary trajectory. This research presents a chromosome-scale assembly and annotation of the *C. megacephala* genome. We further conducted evolutionary analyses of gene families and curated those implicated in chemosensation and detoxification pathways. These genomic resources provide foundational insights into the species’ adaptive mechanisms, offering new tools for forensic entomology research.

**Abstract:**

*Chrysomya megacephala*, as one of the common blowflies, displays biological characteristics, such as ovoviviparity and carrion-feeding adaptation. Thus, this species is generally considered of significant ecological, medical, and forensic importance. However, without a high-quality pseudo-chromosome genome for *C. megacephala*, elucidating its evolutionary trajectory proved difficult. Herein, we assembled and analyzed a high-quality chromosome-level genome assembly of the *C. megacephala*, combined with PacBio HiFi long reads, Hi-C data, and Illumina reads. The pseudo-chromosomes assembly of *C. megacephala* spans 629.44 Mb, with 97.05% anchored to five chromosomes. Final assembly includes 1056 contigs (N50 = 1.68 Mb), and 97 scaffolds (N50 = 121.37 Mb), achieving 98.90% BUSCO completeness (*n* = 1367). Gene annotation predicted 17,071 protein-coding genes (95.60% BUSCO completeness), while repeat masking identified 244.26 Mb (38.82%) as repetitive elements. Additionally, 3740 non-coding RNAs were characterized. Gene family analyses resulted in 10,579 gene families, containing 151 gene families that experienced rapid evolution. Comparative genomic analyses showed that the expanded genes are related to reproduction and necrophagous habits. In addition, we annotated the gene family P450s, CCEs, IRs, GRs, and ORs, all of which represent remarkable expansion, playing a crucial role in the mechanism of locating the hosts for forensic insects. Our research establishes a high-quality genome sequence to facilitate subsequent molecular investigations into significant species within forensic entomology.

## 1. Introduction

The accurate estimation of the postmortem interval (PMI) is a major factor in criminal investigative directions within criminal cases and remains a challenging and hotly debated topic in forensic research [1]. Forensic entomology, which uses insects and other arthropods for forensic investigations, provides validated methodologies for estimating not only time since death, but also location, cause of death, and even evidence of antemortem trauma or abuse [2,3,4,5]. Among the insects utilized, blow flies (Diptera: Calliphoridae) are paramount, typically serving as primary colonizers of vertebrate carrion [6]. Their ecological role as necrophagous decomposers, coupled with an exceptionally acute olfactory system, drives females to rapidly locate and oviposit on remains, often initiating colonization within mere hours postmortem [6,7,8,9]. Consequently, blow fly larvae constitute frequent and critical entomological evidence in death investigations [6,7].

*Chrysomya megacephala* is a forensically significant calliphorid species, exhibiting nearly ubiquitous distribution across terrestrial ecosystems [10,11,12]. Due to its unique feeding habits, this species is frequently ranked as the most prevalent insect encountered in homicide investigations [7]. Under favorable environmental conditions, female flies can arrive at a corpse within 2 h after death and lay 220–325 eggs [12,13], which offers a large amount of available data for forensic investigations, especially cases involving indoor death scenes [4]. This predictable colonization behavior generates crucial temporal data for PMI estimation, proving particularly valuable in complex scenarios involving indoor death scenes [4].

In addition, *C. megacephala* is also an important coprophagous insect, usually found in rural latrines and livestock manure piles [14]. The coprophagous traits make this species an excellent research model for the bioconversion of human excrement, animal manures, and other organic waste [14,15]. Previous studies have shown that its larvae can rapidly and efficiently process food waste within 5–6 days without secondary pollution [14,15,16].

The complicated feeding habits of this species heavily rely on volatile organic compounds (VOCs) for carcass location and oviposition site selection, and excellent detoxification metabolic capacity [17,18]. Key carrion-associated VOCs like dimethyl disulfide and dimethyl trisulfide serve as critical chemosensory cues for necrophagous insects [18]. This chemosensory process is mediated by peripheral proteins, including odorant-binding proteins (OBPs), odorant receptors (ORs), ionotropic receptors (IRs), and gustatory receptors (GRs) [19,20]. Concurrently, the ability to thrive in decomposing remains and resist insecticides of insects is closely linked to its detoxification capacity [21,22]. The cytochrome P450 (CYP) superfamily [23] catalyzes the Phase I oxidative metabolism of diverse xenobiotic and endogenous substrates. Similarly, carboxyl/cholinesterase (CCE) enzymes [24] hydrolyze carboxylic esters, serving as primary detoxifiers of dietary and environmental toxins. Although these detoxification and chemosensory systems are well conserved among insects, the molecular basis of these gene families in *C. megacephala* remains insufficiently characterized.

Despite *C. megacephala* holding forensic and bioconversion significance, the scarcity of high-quality genomic resources has limited exploration of its evolutionary adaptations. The absence of chromosome-level resolution further restricts comparative analyses of chemosensory-driven behaviors like host location. Herein, we present a chromosome-resolved genome assembly through integration of Illumina short reads, PacBio HiFi long reads, and Hi-C data. We further annotated key detoxification (P450s, CCEs) and chemoreception (IRs, GRs, ORs) gene families to elucidate evolutionary mechanisms underlying host selection and feeding ecology. This genome provides an important resource for the host selection and adaptability evaluation of forensic insects.

## 2. Materials and Methods

### 2.1. Taxon Sampling and Sequencing

All samples of *C. megacephala* were collected from Jinan, Shandong, China (36°39′15″ N, 117°03′17″ E, 84 m) in June 2023 by D.Z. and L.L. Samples underwent immediate deposition in liquid nitrogen and this was followed by −80 °C storage before DNA/RNA extraction and sequencing. D.Z. and L.L. integrated morphological and molecular characteristics to identify samples. We used thorax tissue (removed all metasoma of the sample to reduce potential contamination from microbes) to extract DNA.

DNA for PacBio HiFi sequencing was prepared using an SMRTbell^®^ Express Template Prep Kit 2.0 (Pacific Biosciences, Menlo Park, CA, USA, 101-853-100), with 15-kb libraries sequenced on the Sequel IIe platform. Parallel Illumina libraries (350-bp inserts) were constructed with a TruSeq Nano DNA HT Kit and sequenced as 150-bp paired-end reads on NovaSeq 6000 (Illumina Inc., San Diego, CA, USA). A Qiagen Blood and Cell Culture DNA Mini Kit (QIAGEN GmbH, Hilden, Germany) was used to extract DNA. RNA extracted via TRIzol reagent was converted to libraries using TruSeq RNA v2 (Illumina Inc., San Diego, CA, USA), while Hi-C sequencing utilized 150-bp paired-end reads on a BGI MGISEQ-2000 platform (BGI Group, Beijing, China).

### 2.2. Genome Assembly

BBTools v38.82 [25] was utilized for quality control of the Illumina data: “clumpify.sh” script was used to remove duplicate reads, and quality trimming was performed by bbduk.sh. In addition, khist.sh was used for K-mer analysis; k-distribution, the heterozygosity, repetitive elements, and the size of *Chrysomya megacephala* were predicted via Genomescope v2.0 [26].

Hifiasm v0.19.8 [27] was performed for the PacBio HiFi long reads primary assembly with the default parameters, and Hifiasm assembly retains only contig sequences with sequencing depths greater than 5X, because low-depth reads are highly likely to be contaminated or erroneous sequences. Purge_Dups v1.2.5 [28] was conducted to remove the heterozygous regions to improve the accuracy of assembly. Juicer v1.6.2 [29] was utilized to align the Hi-C reads to the assembly. Chromosomal anchoring of primary contigs was performed with 3D-DNA v.180922 [30]. MMseqs 2 v11 [31] was performed to identify the potential contaminants by querying the UniVec and NCBI nucleotide databases with a minimum sequence identity threshold “--min-seq-id” of 0.8. Genome completeness was assessed via BUSCO v5.4.4 [32] using the insecta_odb10 (*n* = 1367) database as the reference. For independent validation, Illumina short reads and PacBio HiFi reads were aligned to the assembly using Minimap2, with alignment statistics derived via SAMtools to quantify read utilization and assembly integrity.

### 2.3. Genome Annotation

Genome annotation encompassed repeat masking, protein-coding gene (PCG) prediction, functional assignment, and non-coding RNA identification. A custom repeat library was constructed using RepeatModeler v2.0.2 [33] with its LTR discovery pipeline (-LTRstruct) for de novo identification, which was based on a specific structure of repeats, and then combined with RepBase-20181026 [34] and Dfam 3.5 [35] databases to establish a custom library. RepeatMasker v4.1.4 [36] subsequently masked repetitive elements against this integrated database. Non-coding RNAs were characterized via Infernal v1.1.4 [37] using the Rfam database, while tRNAscan-SE v2.0.9 [38] curated transfer RNAs under ”EukHighConfidenceFilter” parameters to exclude low-confidence predictions.

Protein-coding gene (PCG) annotation integrated three complementary approaches: ab initio prediction, transcript evidence, and homology inference, implemented through MAKER v3.01.03 [39]. BRAKER v2.1.5 [40] trained Augustus v3.3.3 [41] and GeneMark-ES/ET/EP v4.48 [42] for ab initio modeling, utilizing RNA-Seq alignments (processed via HISAT2 v2.2.0 [43]) and OrthoDB v11 [44] reference proteins. Transcript-based predictions employed StringTie v2.1.4 [45] for RNA assembly, while homology detection leveraged cross-species protein alignments. For homology-based prediction, genes were predicted using GeMoMa v1.9 [46] through protein homology and intron location analysis, applying the parameters “GeMoMa.c = 0.4 GeMoMa.p = 10.”, and we aligned homologous proteins of five species *Anopheles gambiae* (GCF_000005575.2), *Aedes aegypt* (GCF_002204515.2), *Sarcophaga bullata* (GCA_005959815.1), *Drosophila melanogaster* (GCF_000001215.4), and *Lucilia cuprina* (GCF_000699065.1) (downloaded from the NCBI database). Finally, the predictions from RAKER and GeMoMa were merged and used as the ab initio input for MAKER.

**Figure 1:**
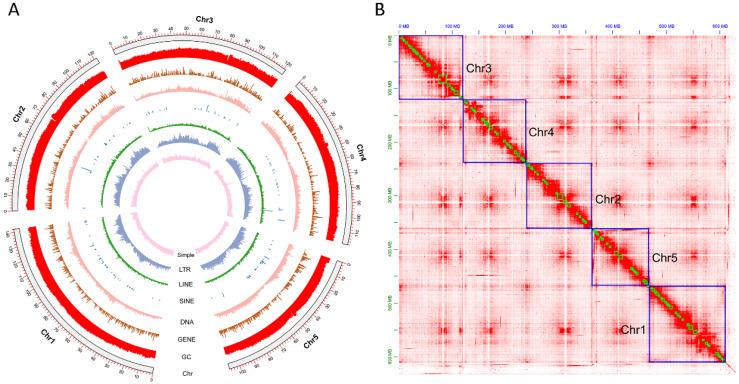
Genome characteristics of *Chrysomya megacephala*. (**A**) *C. megacephala* genome architecture visualization; (**B**) Whole-genome Hi-C contact map of *C. megacephala*.

To functionally annotate the PCGs, Diamond v0.9.24 [47] was performed to search against the UniProtKB (SwissProtTrEMBL) database with a sensitivity model. InterProScan 5.41-78.0 [48] was employed for database searches, including Pfam [49], Smart [50], Superfamily [51], CDD [52], and Gene3D [53]. eggNOGmapper v2.0.1 [54] was performed to search the eggNOG v5.0 database to assign the protein domains, Go terms, and pathways (KEGG, Reactome). TBtools v 0.665 [55] was utilized to generate the visual of *C. megacephala* genomic characters, containing LTR, SINE, Chr, LINE, DNA, GC, and GENE (Figure 1A).

### 2.4. Phylogenomics and Gene Family Evolution Analyses

OrthoFinder v2.5.2 [56] was performed to identify the orthology of PCG sequences, based on protein annotation sequences of 10 Diptera insect species downloaded from NCBI: one Sarcophagidae (*Sarcophaga bullata* [GCA_005959815.1]), two Culicidae (*Anopheles arabiensis* [GCF_016920715.1], *Aedes aegypti* [GCF_002204515.2]), two Tephritidae (*Bactrocera oleae* [GCF_042242935.1], *Ceratitis capitata* [GCF_000347755.3]), one Drosophilidae (*Drosophila melanogaster* [GCA_029775095.1]), two Calliphoridae (*Lucilia cuprina* [GCA_022045245.1], *Chrysomya megacephala* [GCA_049350715.1]), one Muscidae (*Musca domestica* [GCF_030504385.1]), and one Muscidae (*Stomoxys calcitrans* [GCF_963082655.1]). Sequence alignments were performed by Diamond with ultra-sensitive mode (“-S diamond_ultra_sens”).

Single-copy orthologs detected by OrthoFinder served as the basis for inferring phylogenetic relationships across 10 Diptera insects. MAFFT v7.450 [57] aligned these sequences under an L-INS-I high-accuracy setting, and TrimAl v1.4.1 [58] subsequently trimmed alignment gaps and unreliable segments. FASConCAT-g v1.04 [59] was applied to concatenate alignments. We used the LG model in IQ-TREE v2.07 [60] to reconstruct the phylogenetic tree with 1000 SH-aLRT [61] and UFBoot2 replicates [62]. Divergence time estimation employed MCMCTree in PAML v4.9j [63] using two fossil constraints from PBDB, which followed previous research [11]: Culicidae (105.91–234.53 Ma), *Stomoxys calcitrans*, and *Musca domestica* (26.97–36.96 Ma) were used for fossil calibration.

CAFÉ v4.2.1 [64] detected expanded or contracted gene families by modeling a stochastic birth–death process (λ) across the provided phylogenomic tree and gene family dataset. Functional enrichment analysis (GO and KEGG) was conducted using R package ClusterProfiler v3.10.1 [65] for protein-coding genes from significantly expanded families.

### 2.5. Detoxification and Chemoreception Gene Family Annotation

We re-annotated five gene superfamilies, including three chemosensory-related (ORs, GRs, and IRs) gene families, and two detoxification-related (CCEs, CYPs) gene families. Gene family Hidden Markov models (HMMs) were downloaded from Pfam: GR (PF08395), OR (PF02949), IR (PF10613), CCE (PF00135), and CYP (PF00067). The protein sequences of *Drosophila melanogaster* were used as reference sequences. BITACORA v1.3 [66] was employed to identify gene families. BITACORA compared the protein-coding genes, which were annotated by MAKER and the genomic assembly results using BLASTP and TBLASTN, respectively, and then HMMER v3.3 [67] was used to identify protein domains of the gene family, with the E-value *1e-5*. Subsequently, IGV v2.14.0 [68] was used to manually correct the boundaries of exon and intron, and filtered out the pseudogenes with stop codons based on the results of transcriptome alignment, BRAKER/GeMoMa prediction, and MAKER prediction. MAFFT was used to align genes with the L-INS-I model, and then trimAl was used to filter the genes, while IQ-TREE was applied to infer the maximum likelihood phylogenetic tree for each gene family.

## 3. Results and Discussion

### 3.1. Genome Assembly

Our sequencing yielded 191.12 GB clean reads in total, including 52 Gb Illumina reads, 25.93 Gb transcriptome data, 26.09 Gb PacBio HiFi reads, and 87.10 Gb Hi-C reads (Table S1). After polishing, redundancy checks, contaminant removal, and Hi-C scaffolding, we obtained the final genome size of *C. megacephala* is 629.44 Mb, and the BUSCO completeness reached 98.90%, containing 1321 single-copy BUSCOs (96.60%), 31 duplicated BUSCOs (2.30%), two fragment BUSCOs (0.10%), and 14 missing BUSCOs (1%) (Table 1). The genome *C. megacephala* included 97 scaffolds and 1056 contigs, with the scaffold/contig N50 size of 121.37/1.68 Mb (Table 1). The assembly exhibited 29.03% GC content (Table 1). In addition, over 97% of the assembly was occupied in 5-pseudo-chromosomes (Figure 1B). The mapping rates of Illumina, transcriptome, and PacBio HiFi reads were 83.40%, 89.63%, and 99.98%, respectively.

### 3.2. Genome Annotation

RepeatMasker analysis revealed that repetitive elements constitute 38.82% (244.26 Mb) of the *C. megacephala* genome (Table 1). The repetitive fraction comprised 2.03% SINEs, 0.63% LINEs, 7.27% LTRs, 3.30% DNA transposons, and 21.98% unclassified repeats (Table S2). The most prevalent DNA transposon was the MULE-MuDR family (1.13%), highlighting its importance in the DNA transposon group (Table S2). Meanwhile, we identified 3740 ncRNAs, containing 636 rRNAs, 78 miRNAs, 972 tRNAs, and 145 snRNAs (Table 1 and Table S3).

The *C. megacephala* genome annotation via MAKER3 identified 17,071 protein-coding genes (PCGs), exhibiting an average gene length of 9950.5 bp. The average number of exons, CDS, and introns per gene was 4.5, 4.3, and 3.4, having mean lengths of 372.8 bp, 2586.3 bp, and 411.7 bp, respectively (Table 1 and Table S4). BUSCO assessment indicated high prediction quality, with 95.60% completeness (*n* = 1367 BUSCOs): 887 single-copy (64.90%) 420 duplicated (30.7%), 3 fragmented (0.20%), and 57 missing (4.20%). Diamond searches matched 15,517 genes to the UniProtKB database. Combining with InterProScan and eggNOG annotation results, we identified 11,412 GO items, 5032 KEGG items, respectively (Table S4).

### 3.3. Phylogenetic and Gene Family Evolution Analyses

**Figure 2:**
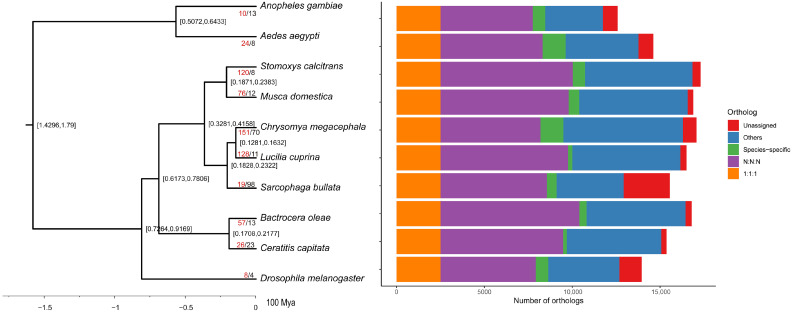
The phylogeny and gene family analyses among 10 Diptera species, and the node value represented the divergence times under the 95% highest probability densities (100 Ma). Terminal labels indicate counts of significantly expanded (red) and contracted gene families.

Gene families across 10 Diptera species were identified using OrthoFinder (see Section 2), yielding 14,928 orthogroups containing 148,537 genes. Classification revealed 1657 species-specific orthogroups (6740), 6564 universal orthogroups, and 2511 single-copy orthogroups (Figure 2; Table S5). For *C. megacephala*, 16,304 genes grouped into 10,484 gene families showed 1630 contracted, 836 expanded, and 151 rapidly evolving families.

Filtering by “symtest” removed 252 loci and resulted in 2259 loci (1,152,909 amino acid sites). All these genes were applied to reconstruct the phylogenetic relationship using IQ-TREE, and the support of all nodes was 100/100, showing that Sarcophagidae is the sister to Calliphoridae, which is a similar finding to a previous study, and the Sarcophagidae and Calliphoridae diverged during Eocene period (32.81–42.58 Ma) (Figure 2).

Among the rapidly evolving gene families identified in *C. megacephala*, several demonstrate critical functional specializations that directly contribute to its ecological success, establishing it as a species of significant importance in forensic entomology. Several of them play important roles in the survival of forensic entomology, including those related to odor-related factors, digestion, and detoxification (Figure S1). Odorant receptors have been found that are associated with feeding behavior and host location, while detoxification appears to be related to the ability to thrive in decomposed corpses [69,70].

**Figure 3:**
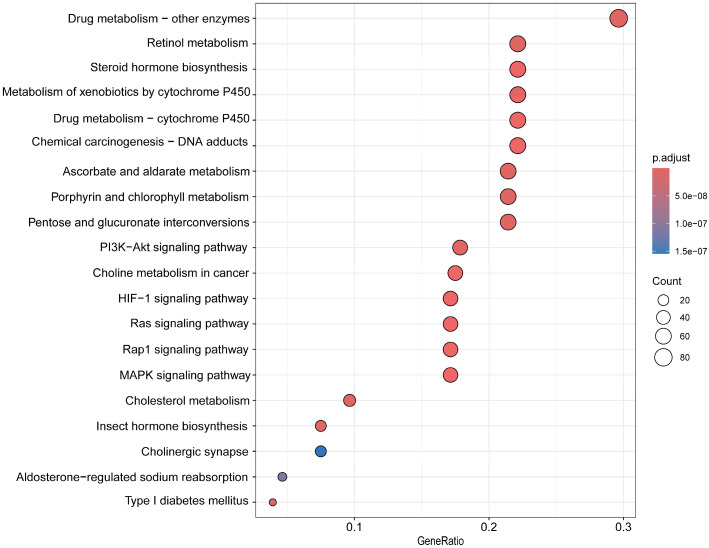
KEGG annotation of the rapid evolution gene families for *Chrysomya megacephala*.

**Figure 4:**
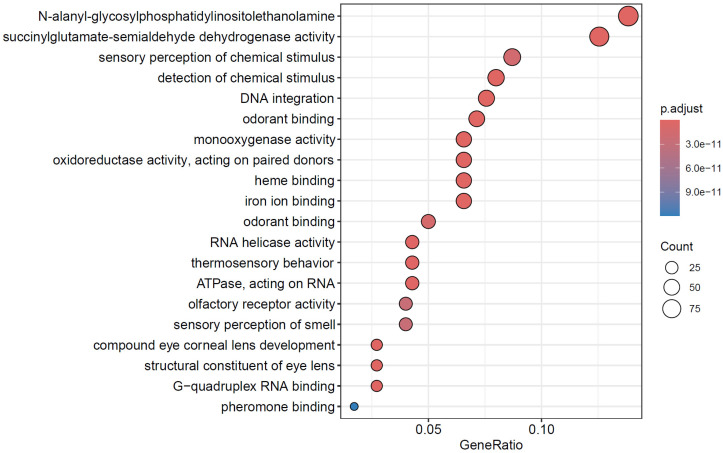
GO annotation of the expanded gene families of *Chrysomya megacephala*.

Our KEGG enrichment analysis revealed significant overrepresentation of pathways related to xenobiotic metabolism and signal transduction, including oxidoreductase activity, dehydrogenase activity, chemical reactions, sensory perception, and detection of chemical stimuli (Figure 3). Notably, several pathways, such as drug metabolism and cytochrome P450 activity, were enriched, highlighting their relevance to detoxification and endocrine regulation. (Figure 3). Similarly, GO enrichment analysis identified major biological functions associated with metabolic processes and hormone biosynthesis, including pathways involved in drug metabolism, retinol metabolism, steroid hormone production, and insect hormone biosynthesis (Figure 4). These enriched functions suggest an enhanced capacity for chemical signal processing and metabolic regulation in *C. megacephala*, consistent with its ecological role in toxic and decomposition-rich environments.

The prevalence of enriched GO/KEGG terms among expanded families suggests pronounced functional specialization in this species. Specifically, the enrichment of detoxification-related pathways reflects adaptive evolution to cope with chemically complex and toxic environments, such as decomposing carcasses [69,70]. Meanwhile, the expansion of chemosensory gene families underpins the species’ reliance on olfactory cues for locating hosts and oviposition sites [70]. The observed enrichment in hormone biosynthesis pathways may further support developmental plasticity and reproductive regulation, essential for rapid colonization and life cycle progression in necrophagous niches [70,71] (Figure 3 and Figure 4).

### 3.4. Detoxification Gene Family Annotation

Given the complex feeding behavior and complex host localization mechanism for *C. megacephala*, we performed manual annotation of shared gene families in this study, including P450s, GRs, CCEs, IRs, and ORs.

**Figure 5:**
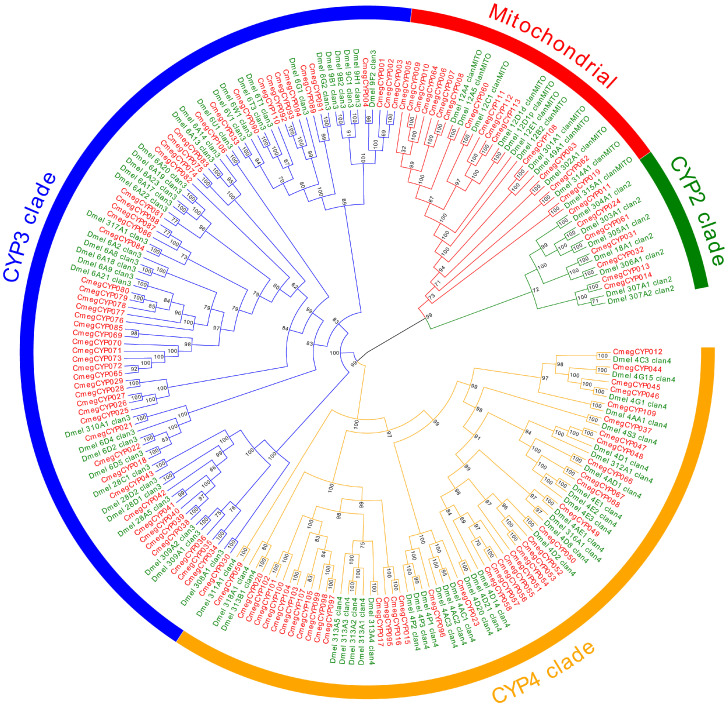
Expansion of the P450 gene family for *Chrysomya megacephala*. The phylogenetic tree illustrates paralogous and orthologous connections between all P450 genes from *C. megacephala* and *Drosophila melanogaster*. Node support shows the bootstrap values.

P450s represent the most functionally significant enzyme superfamily for xenobiotic metabolism in arthropods [72]. For the genome *C. megacephala,* we identified 113 CYPs genes, including four main classes: CYP2 (7), mitochondrial P450 (15), CYP3 (52), and CYP4 (39) (Figure 5; Tables S6 and S7). Compared with *D. melanogaster*, we found significant expansions in the CYP3 and CYP4 clades within *C. megacephala*. These clades have critical detoxification functions through the biotransformation of endogenous and xenobiotic compounds in intestinal and malpighian tubule tissues, providing cellular protection against toxicants [73,74], and likely represent lineage-specific adaptations to chemically complex carrion environments of *C. megacephala*. These pathways confer unique biological capabilities, including insecticide resistance and tolerance to highly toxic decomposing environments. This is exemplified by the significant expansion of P450 genes, strongly associated with the efficient neutralization of hazardous metabolites (e.g., putrescine, cadaverine, and necromones) produced during microbial decomposition and tissue breakdown, enabling the organism to thrive in carrion [72,73,74].

The CCEs superfamily represents functionally diverse carboxylic ester hydrolases; genes of this superfamily have been found that associate with the endogenous compound homeostasis (hormones, pheromones, acetylcholine) and xenobiotic detoxification [21]. For the genome *C. megacephala*, we identified 61 CCEs. Notably, the number of CCEs is remarkably conserved in *D. melanogaster*, but we found a clear expansion in the *C. megacephala* (Figure S2; Table S8). This suggests that *C. megacephala* may have evolved a more versatile enzymatic repertoire to cope with diverse chemical exposures during carrion colonization.

### 3.5. Chemoreception Gene Family Annotation

Insects depend critically on olfactory signals to navigate their chemical surroundings, employing specialized gene families to sense diverse volatile molecules [19]. GRs, IRs, and ORs constitute the primary chemosensory systems orchestrating essential survival responses: initial odor detection, targeted movement, specific signature identification (e.g., food sources, mates), and subsequent feeding or egg-laying decisions [21,69].

**Figure 6:**
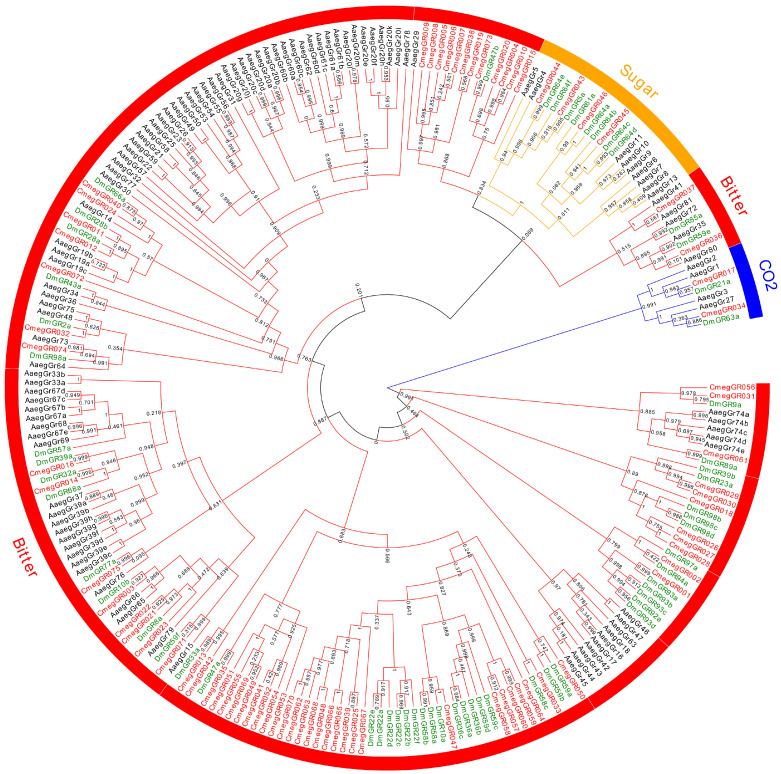
The phylogenetic relationship of the *Chrysomya megacephala* GR gene family, with nodal bootstrap values annotated.

Our systematic cataloging in the forensic fly *C. megacephala* identified 75 GRs, 91 ORs, and 134 IRs (Figure 6, Figures S3 and S4; Tables S9–S11). This quantification reveals a markedly amplified chemoreceptor repertoire relative to *D. melanogaster*. These expansions likely reflect enhanced olfactory and gustatory sensitivity, which is essential for detecting volatile organic compounds and selecting suitable oviposition sites. *C. megacephala* expanded the GR, IR, and OR gene family, enhancing its olfactory detection range. This broadened chemosensory capacity facilitates adaptation to varied niches, especially decomposition environments, and enables precise identification of critical resources like carrion in distinct decay phases [19,20,69,70,71]. This genetic basis directly underpins the fly’s exceptional proficiency in swiftly locating ephemeral cadavers (crime scenes), cementing its forensic role as a primary colonizer [69,70,71]. Characterizing these amplified gene sets is vital for deciphering the molecular basis of olfaction in forensic flies. Furthermore, these pathways provide a foundation for elucidating the molecular mechanisms of olfaction in forensic fly species, thereby enhancing our understanding of the PMI. Unfortunately, functional validation was not conducted in this study. Nevertheless, the comprehensive annotation results generated here offer important targets and hypotheses for future functional studies.

## 4. Conclusions

Here, we successfully assembled a high-quality chromosome-level genome of an important forensic entomology *C. megacephala*. The final assembly was 629.44 Mb with BUSCO 98.90% completeness, including 1321 (96.60%) single-copy BUSCOs, 31 (2.3%) duplicated BUSCOs, and 14 (1.00%) missing BUSCOs. Comparative genomics research found that the chemosensation (IRs, ORs, and GRs) and detoxification (P450s, CCEs) represented a remarkable expansion, indicating that these gene families play a vital regulatory role in diverse feeding behavior and host localization for forensic entomology. This high-quality genome resource advances the understanding of evolutionary adaptations and molecular mechanisms for this species while establishing essential datasets for comparative genomic research. Although functional validation was not performed, this genome provides a valuable resource for future studies on ecological adaptation and forensic application. It establishes a foundation for exploring gene functions related to xenobiotic metabolism, olfaction, and potentially improving tools for postmortem interval (PMI) estimation.

## Figures and Tables

**Table 1:** Genome assembly and annotation statistics for the chromosome-level assemblies of *Chrysomya megacephala*.

Characteristics	*C. megacephala*
Genome assembly	
Size (Mb)	629.44
Number of scaffolds	97
Number of chromosomes	5
Scaffold N50 length (Mb)	121.37
GC (%)	33.74
BUSCO completeness (%)	98.90
Protein-coding genes	
Gene number	17,071
Mean gene length (bp)	9950.5
BUSCO completeness (%)	99.00
Repetitive elements	
Size (Mb)	244.26 (38.32%)
DNA transposons (Mb)	20.96 (3.30%)
SINEs (kb)	56.23 (0.00%)
LINEs (Mb)	12.77 (2.03%)
LTRs (Mb)	45.85 (7.27%)
Unclassified (Mb)	138.36 (21.98%)
Number of ncRNA	3740
rRNA	636
miRNA	78
snRNA	145

## Data Availability

The data are contained within the article or Supplementary Materials. All raw data and the genome of *C. megacephala* have been successfully deposited at NCBI database under the Project PRJNA1121482. Annotation of repetitive elements and other gene predictions results are submitted in the Figshare database: https://figshare.com/account/home#/projects/243644 (accessed on 18 July 2025).

## Supplementary Materials

The following supporting information can be downloaded at: https://www.mdpi.com/article/10.3390/biology14080913/s1, Table S1. Statistics of sequencing data for *Chrysomya megacephala*. Table S2. Repeat annotation in the *Chrysomya megacephala* genome assembly. Table S3. Annotations of non-coding RNAs in the *Chrysomya megacephala* genome assembly. Table S4. Gene annotation results of *Chrysomya megacephala*. Table S5. Statistics of gene families. Table S6. Gene quantity of different gene families. Table S7. Gene information of CYP. Table S8. Gene information of CCE. Table S9. Gene information of GR. Table S10. Gene information of IR. Table S11. Gene information of OR. Figure S1. Twenty significantly expanding gene families of the *Chrysomya megacephala* genome; Figure S2. The phylogenetic relationship of the *Chrysomya megacephala* CCE gene family, with nodal bootstrap values annotated; Figure S3. The phylogenetic relationship of the *Chrysomya megacephala* IR gene family, with nodal bootstrap values annotated. Figure S4. The phylogenetic relationship of the *Chrysomya megacephala* OR gene family, with nodal bootstrap values annotated.
