# Supplementary material for: A High-Quality Chromosome-Level Genome Assembly and Comparative Analyses Provide Insights into the Adaptation of Chrysomya megacephala (Fabricius, 1794) (Diptera: Calliphoridae)
Source: Biology (Basel). 2025 Jul 22;14(8):913. doi: 10.3390/biology14080913 (PMC12383674; doi:10.3390/biology14080913)
Supplement: Supplementary file 1 [file biology-14-00913-s001.zip › biology-3736216-supplementary/supplementary figures.pdf]

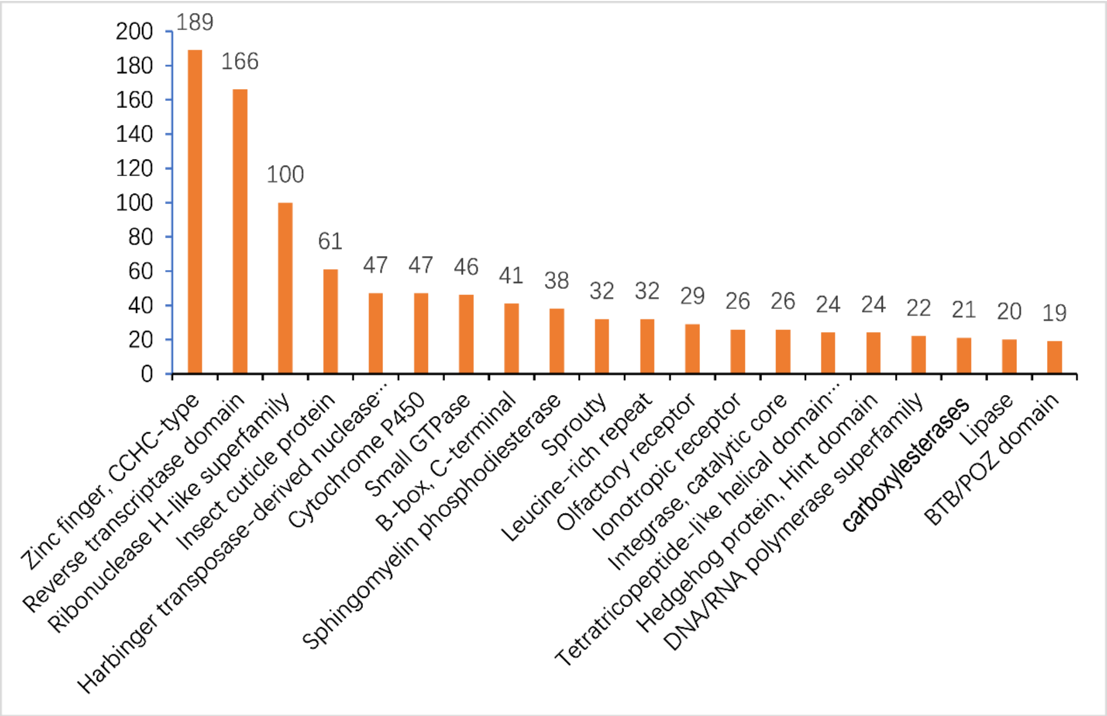

Figure S1. Twenty significantly expanding gene families of the *Chrysomya megacephala* genome

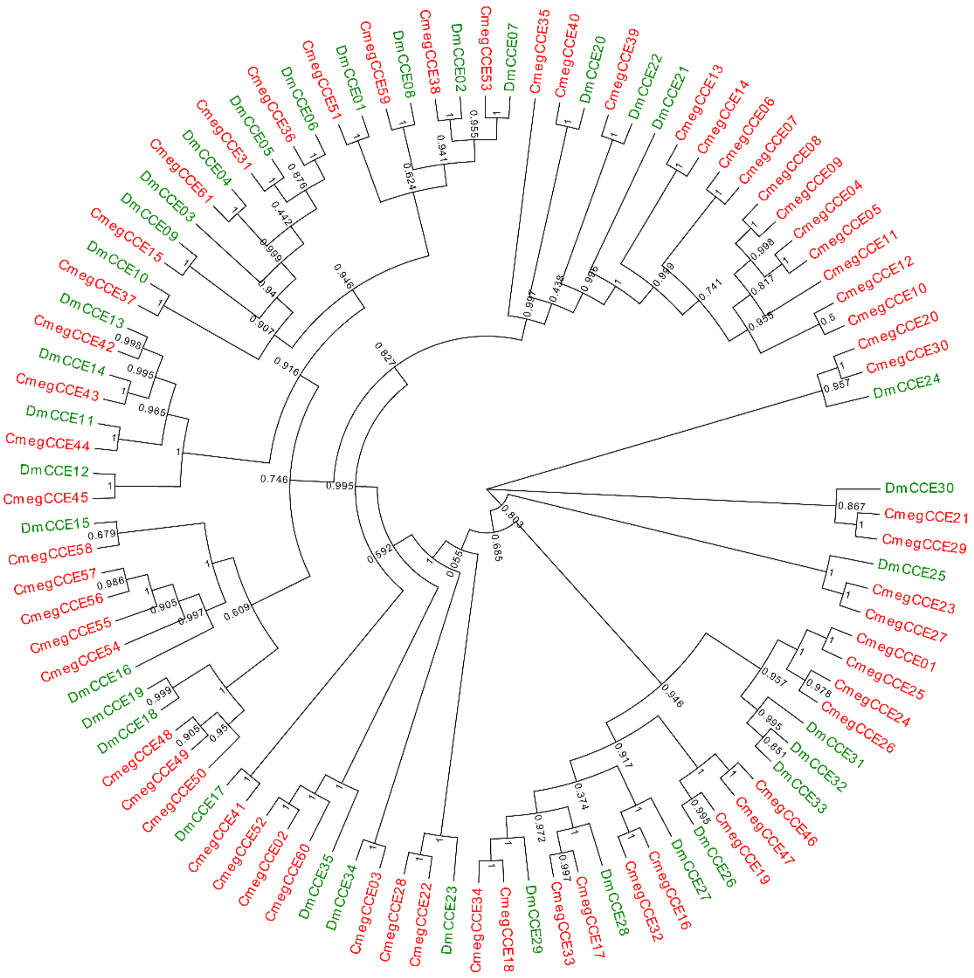

Figure S2. The phylogenetic relationship of *Chrysomya megacephala* CCE gene family, with nodal bootstrap values annotated.

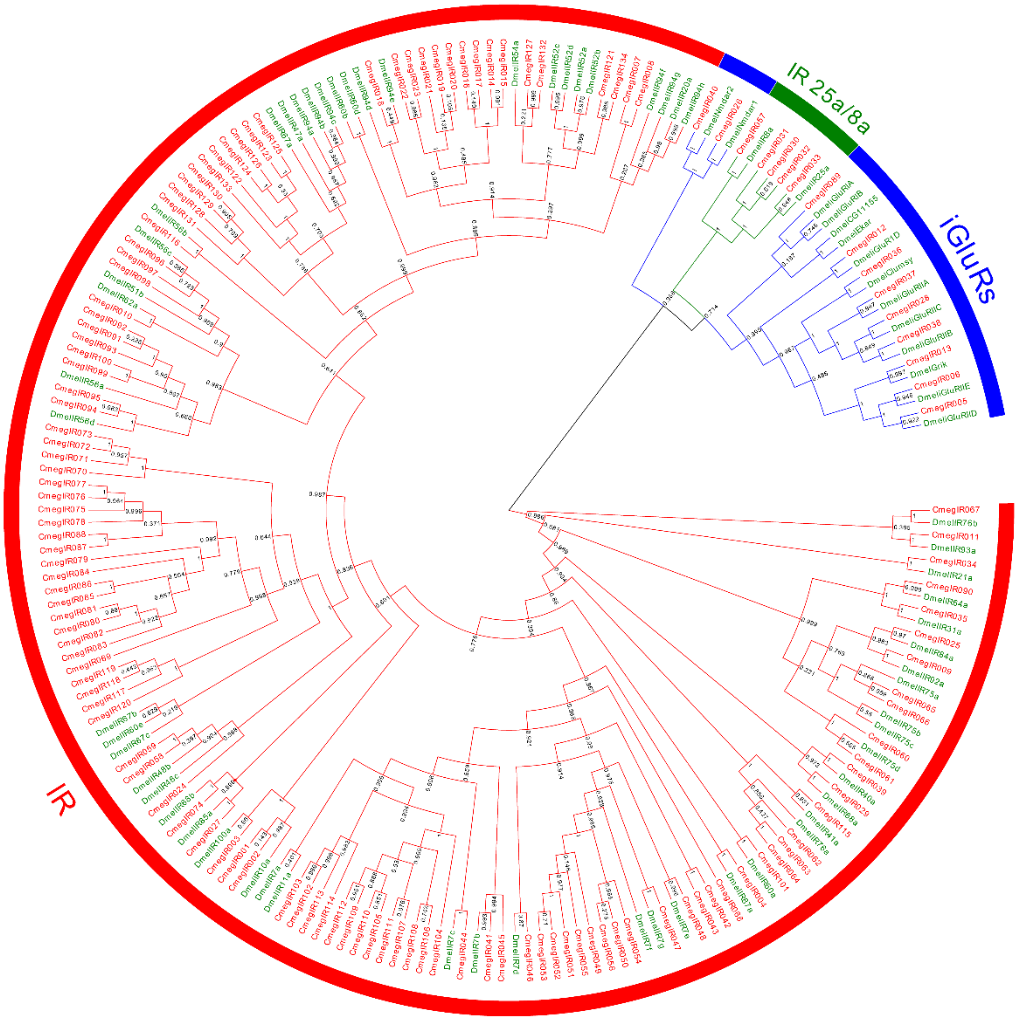

Figure S3. The phylogenetic relationship of *Chrysomya megacephala* IR gene family, with nodal bootstrap values annotated.

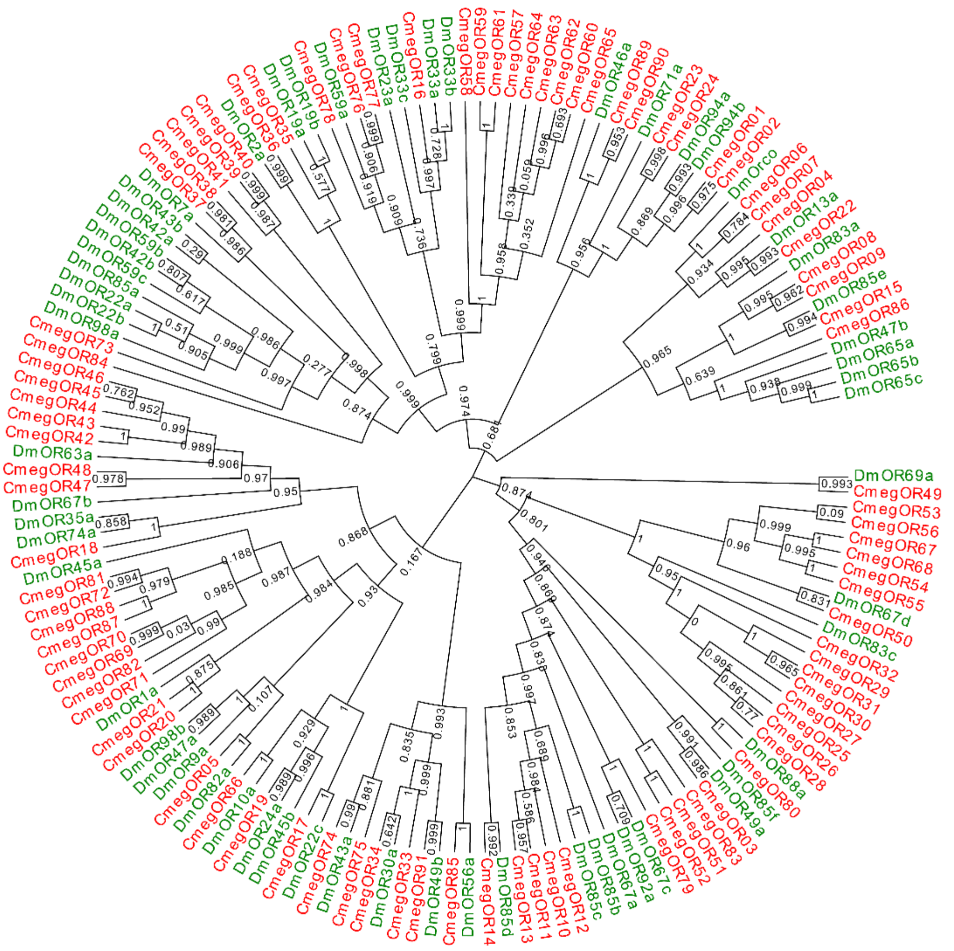

Figure S4. The phylogenetic relationship of *Chrysomya megacephala* OR gene family, with nodal bootstrap values annotated.
